# Sustainable Future Protein Foods: The Challenges and the Future of Cultivated Meat

**DOI:** 10.3390/foods11244008

**Published:** 2022-12-11

**Authors:** Karolina A. Chodkowska, Karolina Wódz, Jakub Wojciechowski

**Affiliations:** 1 Krzyżanowski Partners Sp. z o.o., ul. Zakładowa 7, 26-670 Pionki, Poland; 2 Laboratory of Molecular Biology, Vet-Lab Brudzew, Turkowska 58c, 62-720 Brudzew, Poland

**Keywords:** cultivated meat, sustainable meat production, animal welfare, cell-cultured meat, alternative proteins, future food, novel food

## Abstract

Global pressure from consumers to improve animal welfare, and reduce microbiological risks or the use of antibiotics pose new challenges for the meat industry. Today’s livestock production, despite many undertaken measures, is still far from being sustainable. This forced the need to work on alternative protein types that come from plants, insects, fungi, or cell culture processes. Due to some technical and legal barriers, cultivated meat is not present on the European market, however, in 2020 it was approved in Singapore and in 2022 in the USA. While the technology of obtaining cell cultures from animal muscles has been known and successfully practiced for years, the production of a stable piece of meat with appropriate texture, taste, and smell, is still a problem for several scientific groups related to subsequent companies trying to obtain the highest quality product, in line with the expectations of customers. Although the work on optimal cell meat production has been going on for years, it is still in an early stage, mainly due to several limitations that represent milestones for industrial production. The most important are: the culture media (without animal serum), which will provide an environment for optimal muscle development, natural or close to natural (but still safe for the consumer) stable scaffolds for growing cells. Here, we review the actual knowledge about the above-mentioned challenges which make the production of cellular meat not yet developed on an industrial scale.

## 1. Introduction

Food and Agriculture Organization (FAO) reports indicate that by the middle of this century the world’s population will increase to nearly 10 billion [1]. Such growth poses a challenge not only in terms of the quantity of food that will be required, but also in terms of its quality and the expectations of consumers, who are more and more aware and have greater access to knowledge (including the scientific one).

Meat is one of the essential components of the human diet as it meets the nutritional needs of being rich in wholesome protein, iron, zinc, B vitamins, vitamin A, and fatty acids. The growing consumption of farm-sourced meat is increasingly seen as the origin of many problems in several areas. Over the last 50 years (until 2018), the needs of the global meat market have tripled, reaching the level of 340 million tons [2], before Covid-19 pandemic which had an incredible impact on consumer trends in many countries for different time periods. However, the direction of meat consumption is not the same everywhere, which is closely related to the economic growth of some areas. During the last few years, the European and North American meat markets, seem to be relatively stable or slightly declining. The slight decrease in meat consumption in these countries is mainly related to the increase in consumer awareness and greater knowledge about healthy eating habits, optimal conditions for keeping animals, and their welfare. Moreover, care for the natural environment also seems to be one of the key factors contributing to the reduction of meat consumption in some consumer groups. It should be emphasized that despite taking multifaceted actions, today’s food production is still not fully sustainable. Animal production contributes to the growing emission of greenhouse gases (GHG), and the demand for feed raw materials destroys sometimes very unique ecosystems, negatively affecting biodiversity [3]. FAO reports indicate that nearly 14.5% of total greenhouse gas emission is related to livestock production. Moreover, GHG from meat production comprised about 54% of total emissions from agriculture during the years 2018–2020. The 5% increase in emissions from the meat sector by 2030 is much smaller than the increase in meat production, mainly due to the increased share of poultry production and the projected increase in meat production from a given livestock. Animal production is also linked to the enormous consumption of fresh water (it is estimated that nearly 23% of the total water consumption is used by animal production), which in turn contributes to the increasing water shortages in some parts of the world [4].

All of the above aspects have forced an active search for alternative solutions that will provide the highest quality meat and its products. Cellular meat seems to be such a solution. A denouement, which combines tissue engineering and cell culture to produce muscle that can be used for food production. This kind of production will be more sustainable, respecting animal welfare, and will allow the reduction or complete elimination of antibiotics. Cell-based manufacturing also reduces the risks of chemical contamination and foodborne diseases and zoonosis (*E. coli*, *Campylobacter* spp,. and *Salmonella* spp.), compared to conventional meat. Moreover, what is crucial, this production somewhat is perversely known as production without the participation of animals (the animal appears only at the beginning, as a donor of cells, and in further stages of production sera of animal origin were eliminated from the media). The production of cellular meat is one of the types of developing cellular agriculture and is, in a way, an alternative to plant-based protein but also for classic meat products. This paper contributes to a growing number of review papers about cultured meat [5,6,7,8].

## 2. History and Future of Cell Meat Production

Although the most intensive development of cellular meat has been in the last 10 years, its beginnings are much older. Interestingly, the first information about cultivated meat was published in 1897 in the book Auf Zwei Planeten [9]. Moreover, the most famous phrase: “We shall escape the absurdity of growing a whole chicken in order to eat the breast or wing, by growing these parts separately under a suitable medium. Synthetic food will, of course, also be used in the future”, related to the future concept of producing unconventional meat, was said by Winston Churchill in 1931 [10,11]. In vitro techniques that cell meat production use today (e.g., growing tissues to heal wounds, stem cells in regenerative medicine) have also a long history, and are related to muscle tissue which was first obtained in 1971 by Russel Ross [12], who cultivated muscle fibers from the guinea-pig aorta. What should be emphasized is that the first cell cultures (1907) were carried out on cell models other than muscle cells. They were frog cells grown by Ross Harrison in a special medium with the addition of lymph. Almost 100 years later, in 1999 Willem van Eelen described and patented [13] the first procedure of tissue engineering for cell-cultured meat production. Nearly, at the same time, in 2001, the National Aeronautics and Space Agency (NASA) during food production research, cultivated goldfish meat (and turkey meat later on) [14] which might be a food product successfully used in space travel. Actually, several companies e.g., Aleph Farms are working on the possibilities of cell culture in space. Based on the information from the official website of Aleph Farms [15], on 8 April 2022 samples of animal cells with the basic equipment they need to grow were transported in a SpaceX rocket to the International Space Station, returned on 24 April 2022 and are actually under detailed analysis. However, it was not until the end of the 20th century that it began to abound in reliable research and implementation work, ending with the development of an effective method of obtaining cellular meat and then a finished product from this kind of unique raw material. One of the first patents (2001) related to this kind of product which involved placing the muscle cells into a collagen matrix, keeping them in a nutrient solution, and inducing them to divide, was described by three Dutchmen—W. Wasterhof, W. van Eelen, W. van Kooten [16,17]. At the beginning of its history, research on cell meat was an interesting topic not only for eminent scientists and investors, but also for representatives of the arts. The bioartist, Oron Catts, showed the frog cell mini steaks at the museum in Nantes. It took place in 2003 as part of the famous Disembodied Cuisine project [18]. The concept of cultivated meat was introduced to a wider audience in the early 2000s by Jason Matheny. He also was the co-author of an article on cell-cultured meat and the founder of New Harvest—in vitro meat research organization [19]. In 2008, at the Norwegian Food Research Institute, In Vitro Meat Consortium, a team of scientists from different countries, organized the first official scientific meeting on cell-cultured meat. A year later, Dutch scientists announced their success in porcine cell-based meat production [20]. An important date in the history of this unique product was the year 2013, when an outstanding scientist, Professor Mark Post produced and presented the first hamburger cutlet grown directly from cells [21]. This date was a breakthrough for the rest of the research teams and companies involved in the development of cell meat. However, the most dynamic development, not only of the research itself, but also for activities in the field of marketing, legislation, and increasing availability of commercial products has been observed in the last 5 years. The emergence of new restaurants where it is possible to try cell meat products is mainly in Asian countries, Israel, and the USA. The first restaurant where customers could compare the taste of a traditional chicken burger to that produced in the laboratory was opened in Tel-Aviv [22,23]. The Year 2020 was also a breakthrough for this kind of production, due to the fact that at the end of this year the first commercial sale of cellular meat took place in the “1880 restaurant”, in Singapore. The meat was created by the American company Eat Just [24]. It was estimated that by 2020 there were 55 main companies related to cell-cultured production. Today, this number increased to almost 100 companies and startups involved in this type of production. Both US and the EU are the leaders of such an investment, however, also companies from Israel and Asia significantly contribute to the development of research and the cell-farming market [25]. What should be emphasized, is the fact that many billionaires are private investors for several projects but also key players in the classic protein market such as Cargill, Tyson Foods, PHW, Migros or Grimaud invest a huge amount of money in this kind of meat production. This, in return, may indicate that the industry is aware of the threats that arise for traditional production and is beginning to look for effective alternatives, which today are a premium product, still unavailable in many countries (for various reasons), but in the near future they will be probably found on store shelves next to products from classic production. When analyzing market data from the last few years, it should be noted that this market is developing very dynamically. A number of industry reports indicate that these products will constitute a significant percentage of the food market, next to classic and plant-based products.

According to available reports, e.g., Markets and Markets [26], the global in vitro meat market is estimated at $214 million in 2025 and in 2032 is expected to reach $593 million, recording a CAGR (Compound Annual Growth Rate) of 15.7% in 2025–2032. Based on the analysis of a number of companies related to the cell meat industry, but also the regulated legal status for the production and sale of this type of product, the US seems to have the best chance to become the leader of the in vitro meat market. The main factors driving consumers to switch from conventional to cultivated meat are: health concerns about the consumption of traditional products, increased investor interest in this kind of product and the potential to provide nutritional value to the product tailored to the needs of consumers [26]. In the USA, Canada, but also EU countries, the markets for alternative protein will develop dynamically due to the fact that the number of consumers declaring themselves as flexitarians, as well as, the number of customers, who are curious and open to innovations in food, is growing. In addition, it is assumed that the overall acceptance of alternative protein sources will also increase [26]. A similar forecast can be found in the June 2021 report from the Facts and Factors [27] market research, according to whose volume and share of revenues from the global livestock market was predicted to increase from about 100 million in 2020 to nearly 250 million dollars by 2026, with a 15.7% annual increase in CAGR for the 2021–2026 forecast period.

There are also analyses indicating the possibility of even faster development of the industry. According to the Allied Market Research report [28], the size of the cultivated meat market was valued at more than 1.6 million dollars in 2021 and it is predicted to reach nearly 2800.1 million dollars until the year 2030.

Observing the development of the cellular meat market, it should be noted that with the successive start-ups dealing strictly with meat production, more and more companies related to dedicated cell lines, fat, and various types of nutrients with and without additives of animal origin, or bioreactors are created. It is also a dynamic development of companies developing scaffolds, but also 3D printing. A separate branch, which also has a chance for great development, is the one related to the sensory aspects of the created products and their texture. Already today, a number of companies on the market that until now offered additives that give a taste, smell, or color to classic food products have expanded their portfolio with those dedicated to in vitro products. Analyzing the dynamics of the development of cellular meat alone, it should be expected that the number of companies.

**Figure 1:**
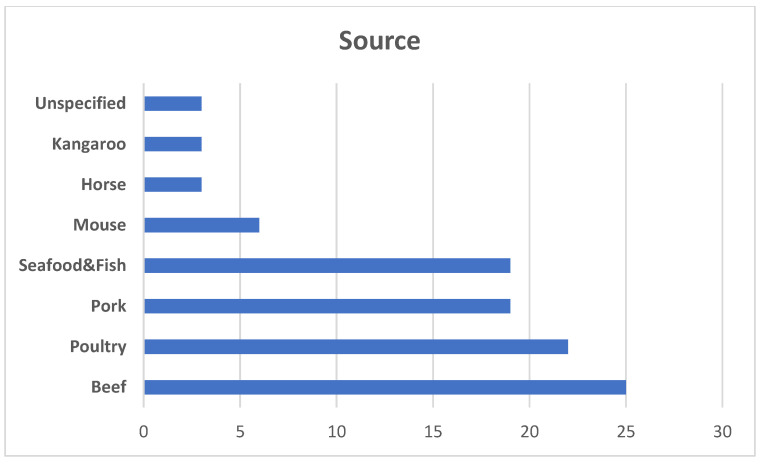
Based on the source global cultivated meat market is divided into four main groups: beef, poultry, seafood, and pork. Other types including mouse, kangaroo, horse, and others (tiger, antelope, etc.) and other rarer species constitute a small percentage so far, which may change in the coming years due to the progress in the technology of culturing these types of cells (based on Choudhury et al. 2020 [30]).

More and more market research indicate that cellular meat will permanently appear not only on store shelves, but also in restaurants. Recent analyzes conducted for Super Meat [29] indicate that laboratory-produced meat products are an interesting diversion for chefs and they will certainly use them when the opportunity arises. More than 250 chefs working in different branches of industry took part in this research. Among this number 86% of participants reported being “interested” in serving cultivated meat and 22% indicated they were “very interested”. Seventy-seven percent of chefs said they would be willing to pay a premium for cultivated meat such as beef and poultry because of their associated benefits in comparison to traditionally reared meat. In the case of an acceptable price for such products, the majority of respondents considered the price higher by 11% and 15% (compared to the classic product) was the most widely accepted by respondents. In the same study, 86 percent of chefs who cook mostly Japanese cuisine reported an increase in demand for meat alternatives. Among Italian chefs, it was only 48%. Meanwhile, when asked what kind of meat they would like to try first, those who cooked mostly American cuisine strongly preferred poultry (64 percent), while those who prepared Italian dishes preferred seafood (56 percent), and Japanese chefs preferred French and Indian exotic meats. It seems that in the case of the US, cellular chicken meat will be the most popular product of this type, as in the case of its traditional counterpart, which is considered the number 1 American fast food. The current global percentage of different types of cellular meat markets based on the source is presented in Figure 1. The poultry segment is increasing during the last two years and is projected to account for the largest share during the forecast period followed by pork. The percentage of seafood/fish will also increase. In addition, the development of technology for obtaining and culturing cells from other, so far less known animal species gives a chance to increase in the future the percentage of the group “others” [30].

**Figure 2:**
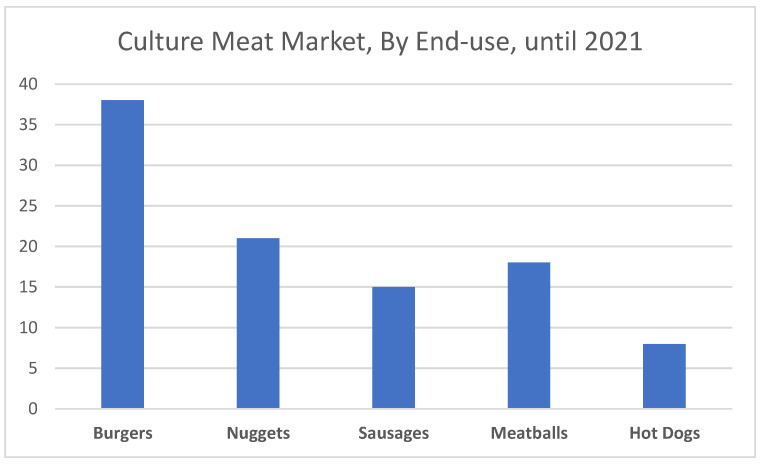
Cultured Meat Market (percentage), By End-use until the end of 2021 (based on reportlinnker.com [31]).

Several market reports divided the cultured meat based on the end use. Generally, at the moment it is segmented into burgers, nuggets, sausages, meatballs, and hot dogs. Based on end-use, the market is segmented into burgers, nuggets, sausages, meatballs, and hot dogs (Figure 2). Despite the emergence of many startups related to other types of meat, it was the burger segment that recorded the highest share in revenues on the farmed meat market in 2021. This is due to increased consumer demand for pure beef in dishes such as burgers. In addition to the products listed above, steaks made of various types of meat (lamb, kangaroo) are increasingly popular, which this year appeared in the portfolio of, among others, companies from Australia [31].

When we analyze the cellular meat market, we cannot focus only on the finished product for humans. An important, although only emerging and currently marginal branch of this industry is pet food, for which cell meat is the raw material. Just as consumers of the food market, owners of dogs and cats are becoming more and more aware. Lots of consumers focus on vegan or vegetarian lifestyles and believe it is more sustainable, they want their pets to follow similar diets. This causes the pet food market to face challenges in terms of sustainable production and the welfare aspect of the animals supplying the raw material. This is additionally supported by a number of pro-animal organizations such as PETA. Wild Earth, Bond Pet Food, Good Dog Food, and Because, Animals are the most well-known companies on the pet food market, which in their portfolio have foods made of cellular meat (e.g., koji) and other alternative proteins (e.g., fungus-based superfood), as well as special treats for cats made of mouse meat. This shows what opportunities the development of the cellular meat market brings, especially in the field of products for animals—a market that is one of the most dynamically developing.

Observing the development of the cellular meat market, it should be noted that along with subsequent start-ups dealing strictly with meat production, more and more companies are created related to dedicated cell lines, fat, and various types of nutrients with and without additives of animal origin, special additives replacing antibiotics or cellular growth stimulators. or finally bioreactors. It is also a dynamic development of companies developing scaffolds, but also 3D printing. A separate branch, which also has a chance for great development, is the one related to the sensory aspects of the created products and their texture. Already today, a number of companies on the market that until now offered additives that give a taste, smell or color to classic food products have expanded their portfolio with those dedicated to in vitro products. Analyzing the dynamics of the development of cellular meat itself, it should be expected that the number of companies will grow, and with them the variety of types of meat produced.

During the preparation of this review, another milestone in the history of cellular meat has been reached. On 18 November, the FDA gave the green light to a cultivated meat product for the first time ever. They evaluated the information UPSIDE Foods submitted to the agency and accepted the firm’s safety conclusion. Moreover, FDA declared that they are ready to work with additional firms developing cultured animal cell food to ensure their food is safe and lawful under the FDA. FDA is actually discussing with several companies representing the cultivated meat sector and in turn, proves that in the near future we can expect more statements of this type, which will significantly affect the development of the cellular meat market, not only in the USA [32].

## 3. Cultivated Meat—Definition, Key Technology Area, and Technical Challenges

### 3.1. Definition and Process of Cellular Meat Production

**Figure 3:**
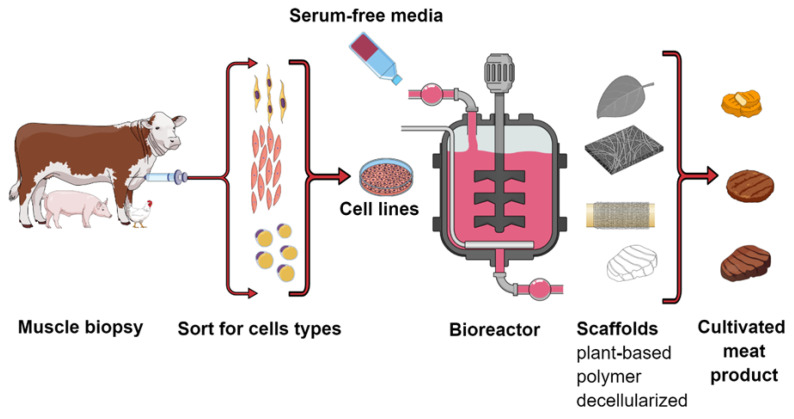
General scheme illustrating the basic steps involved in the production of cultivated meat. 1. Muscle biopsy from selected animals (under anesthesia). 2. Cells selection and cell culture. 3. Harvesting in bioreactors using scaffolds technique. 4. Final cultivated products.

Cultivated meat also known as cultured or in vitro meat, is a product received from isolated muscle cells, which are cultured as cell lines and then, placed in a bioreactor (Figure 3). The goal of the industrial production of cultivated meat is to make a relatively inexpensive meat substitute with the texture and organoleptic properties of real meat. In addition, it is increasingly emphasized that this type of production is environmentally friendly (reducing the carbon footprint) and reduces animal suffering (avoiding the medium and additives used in breeding, the production of which was necessary for the animal).

In the early 2000s, a NASA-funded university group [14] and a group of bio-artists from the Tissue Culture and Art Project [19] produced a small amount of muscle tissue. The NASA team performed a smell test to evaluate tastiness, and the bio-arts group realized the taste-check as part of an art performance. Producing cultured meat based on taking cells from living animals using a biopsy procedure [21], to produce edible tissue with minimal quantities of animal cells compared to livestock methods. Different stem cell types can be used to cultivate meat e.g., skeletal muscle (satellite cells), adipogenic or mesenchymal. Primary muscle-resident progenitor cells isolated from skeletal muscle differentiated into smooth and skeletal muscle, whereas satellite cells only to a skeletal muscle. Differentiation is based on the usage of biological or chemical stimuli present in cell culture media. Collection of the cells can be performed by a biopsy performed on living animals to obtain mature stem cells with limited differentiation potential. Moreover, cells may be acquired by biopsying a recently slaughtered animal where the tissue is still viable, which could be significant from the point of view of religious beliefs (e.g., halal, kosher). An alternative option is to use induced pluripotent stem cells (iPSCs) and embryonic stem cells (ESCs) with an unlimited ability to differentiate into various cells. Pluripotent stem cells first need to be purified or sorted to become enriched and next differentiated into muscle-resident progenitor cells. Similarly, mesenchymal stem/stromal cells (MSCs) and fibro/adipogenic progenitors (FAPs), based on markers CD (cluster of differentiation) expressed by these cells. MSCs express CD73, CD90, and CD105 with a lack of CD34, CD45, and human leucocyte antigen DR (HLA-DR antigens). FAPs express CD90, CD140a, and (stem cell antigen-1) Sca-1.

The challenge of producing in vitro meat is to imitate the muscle-growing environment present in a living organism and repeat it in a laboratory and after in a plant. Typically, the process of producing cultivated meat focuses on the culture of myocytes. However, to obtain muscle tissue that has the potential to imitate meat, multiple cell types are needed (e.g., adipocytes). The technical rules for producing cultured meat are the same as in cell culture but the scale is greater and the product must be inexpensive. To scale up, the development of new pluripotent cell lines from livestock animals or improving the proliferative ability of mature stem cells is needed.

The bioprocess consists of: obtaining the cells, their expansion, and differentiation, and manufacturing the product. The maximum density of proliferating cells is a crucial parameter for the production of in vitro meat. More cells might grow in the same volume of medium in a bioreactor. One of the limitations is metabolite generation and reduction of oxygen availability observed in density > 10^9^ cells/mL [33]. For the production of in vitro meat at a large-scale, about 8 × 10^12^ cells are needed to obtain 1 kg of muscle cells in a 5000 L stirred bioreactor. In addition, muscle cells can expand approximately 20 times under ideal growth conditions. Thus, a significant amount of myocytes can be obtained from a single isolated cell and produce as many cells as possible on a commercial scale. Bioreactors used in cultivated meat production enable the biological process of proliferation of cells in a well-controlled (temperature, pH, oxygen supply, hypoxia, metabolite removal, etc.). The automatization of this process guarantees a high degree of repeatability, and reproducibility, a requirement for large-scale production [34].

Another crucial parameter for commercialization is a sufficient volume of bioreactors for suitable proliferation, differentiation, and growth of cells [16]. A US start-up—Eat just originally used 10,000 L volume bioreactors to produce cultured meat for chicken nuggets and now is starting to use 50,000 L for the commercialization of these methods. The bioreactor facilitates cell suspension, and creates a beneficial environment for the growth of cells, similar to primary tissue, leading to increased yield [35]. The production time depends on the rate of proliferation, maturity of cells, and bioavailability of the nutrient from the culture medium. Cultured meats differ from ordinary meats in terms of color, look, and structure. Generally, they are more yellowish than pink and less red due to lower myoglobin content and higher oxygen saturation [36]. Therefore, to improve the optical impression and flavor of cultured meat, bioprocessing methods should be developed. One of the solutions is culturing in hypoxia conditions for obtaining a generation of cells with higher myoglobin content [37]. Another solution is to add heme from a plant source and hemoglobin or myoglobin from an animal source [38]. Through modification in cultured meat, composition, quality, flavor, fat, and saturated and unsaturated fatty acids the content might alter.

### 3.2. Growth Media for Proliferation and Maturation

One of the major costs of cultured meat is cell culture media, which is currently estimated for 55% to up to over 95% of the product’s costs [39]. Mosa Meat, in 2020 removed fetal bovine serum from the growth medium, which led to an 88-time reduction in production cost. Future Meat Technologies also used animal-free growth with the addition of different plant proteins.

Growth of the cells in meat culture needs nutrients, similar to the environment of tissue that involves proliferation, differentiation, and maturation growth factors. Furthermore, carbohydrates, lipids, sugars, amino acids, minerals, and vitamins or hormones (e.g., insulin). Growth medium should also be xeno-free and chemically well-defined. Muscle stem cells (such as satellite cells and mononucleate myoblasts) are cultivated in a medium containing foetal, calf, horse, or other serum, but the exact composition of it is not specified. In vitro cell cultures grow in sterile media, due to the risk of contamination which can result in bacterial (e.g., Mycoplasma), fungal or viral contamination, and lead to cell death. Antibiotics (e.g., gentamicin, penicillin) are added to the growth media to prevent bacterial infection, and sodium benzoate to yeast and fungus. The main goal of manufacture production cultivated is removed from growth medium antibiotics, hormones, and animal-origin agents, such as serum. Generally, culture medium composition is similar for different species and cell types. For adult stem cells, basal medium, such as Dulbecco’s Modified Eagle Medium (DMEM)/F12 include L-glutamine, non-essential amino acids, and a low concentration of FGF-2. For pluripotent stem cells, the medium formulation is similar but enriched with growth factors such as FGF-2, epidermal growth factor (EGF), transforming growth factor-β (TGF-β), heparin, serum or extracellular matrix components. Additional components may also be used for adult stem cells to improve cell growth.

Optimum culture medium should enhance the proliferation of progenitor or stem cells and their differentiation into myocytes, adipocytes, and connective tissue. Typically, cultured cells are carried out in media containing unspecified humoral components, such as serum, which contain hormones, growth factors, and other proteins, crucial for cell survival, proliferation, and differentiation. The media used for muscle cell growth is most often supplemented with fetal calf serum (FCS) or bovine serum albumin (BSA) at the differentiation stage, thus the problem with non-species proteins and growth factors. Additionally, serum removal from the culture medium terminates the differentiation of muscle progenitor cells into mature myocytes thus serving as a stage cell controller. FBS is an animal-obtained ingredient, making its use contradictory to the motivations for in vitro meat that is not based on extensive livestock farming. Moreover, it is associated with the risk of contamination by viruses or prions [40]. In 2011 Tuomisto et al. developed culture medium base on algae [40], in 2000 Shiozuka and Kimura base on serum-free media with supplementary proteins [41]. For proliferation and differentiation of chick primary myogenic cells, insulin, transferrin, albumin and fibroblast growth factor-2 (FGF-2) as addition to Dulbecco’s modified Eagle minimum essential medium were used [42]. For mature stem cells, basic medium Dulbecco’s Modified Eagle Medium (DMEM)/F12, includes L-glutamine, non-essential amino acids, and a low concentration of FGF-2 were used. To date, free-serum medium TeSR™, and FBM™ were developed for bovine myoblasts and satellite cells [43], B27™ and XerumFree™ additives, and Essential 8™ medium.

Further development of serum-free media from culturing process is set to diminish dependency on animal ingredients and create well-defined chemically, and xeno-free media. Another problem for scaling up is decreasing the cost of the medium, supplements, and especially growth factors. Finding an alternative method to replace growth factors remains an attractive way to significantly diminish the cost of cultivated meat on a large scale [44]. Another method to decrease the cost of production is recycling the medium and its conditioning. Moreover, conditioned medium includes valuable cellular metabolites, extracellular or signaling proteins, cytokines, and growth factors [45], which support cell proliferation and differentiation when mixed with fresh medium [45].

### 3.3. Source of Cells and Bioprocessing Strategies

An interesting approach for in vitro meat is using cell lines, these well-known (e.g., chicken skeletal muscle cells, e.g., LSN Cell Line, Kerfast, steam cells, or new immortalized cell lines. To date, no commercial cell lines for in vitro meat are available, both to laboratory researcher scale and product manufacture. For cultivated meat satellite cells and adipose-derived stem cells are the basis for receiving skeletal muscle and adipocyte. In many cells’ collections, a similar myoblast cell line is unavailable, but its origin is not acceptable for consumers, e.g., rat, mice (L6 and L8 and C2C12, respectively), or hamster. Moreover, meat growing from cell lines should be tasty, nutritious, has an attractive texture and be food-safe for consumers. Additionally, new cell lines used in in vitro meat should evolve from the species well-known to consumers, such as chicken, turkey, duck, geese, cattle, or swine. Ideal cells to manufacture in vitro meat should have a great self-renewing ability and can infinitely continue to divide. In living organisms, myogenesis starts in the stage of formation of the embryo, continues foetus life, and is completed at birth, when myocytes and the muscle tissue are fully mature and developed. In animal skeletal muscle some cells, named myosatellite stem cells, are commonly in a quiescent state (non-dividing). Myosatellite cells start dividing during muscle regeneration after injury or as an adaptation to workload. Another type of steam cells is adult stem cells, in fully developed tissue, which can generate cell types specific to the tissue from which they are derived, but its divisions are limited to 50 to 60 times. For comparison, myosatelite stem cells obtained by biopsy from an adult living animal can divide approximately 20 times. Embryonic stem cells would be an ideal origin for in vitro meat production due to their pluripotent features, but the proliferation and differentiation can be difficult and the source of cells is controversial for consumers. Nevertheless, stem cells are the best candidate to use as a starting cell source due to their self-renewal and differentiation into mature cell types. Undifferentiated progenitor cells such as mesenchymal stem/stromal cells (MSCs), and fibro/adipogenic progenitors (FAPs) placed in organs and tissues have the ability to differentiate into adipocytes, chondrocytes, and fibroblasts, important components of meat. MSCs are usually obtained from the bone marrow and rarely from skeletal muscles, while FAPs are from the interstitial space of skeletal muscle.

Cultivated meat production is based on two methods: self-organizing and scaffolding. In the first method, cells isolated from animals develop into structured meat using a self-organizing process. In the second one, cells differentiate and grow to myoblasts in the scaffold in growth media, and develop into unstructured and soft tissue [46]. In the self-organizing method, cells proliferate in culture media land forming tissues similar to conventional meat in texture, composition, and organoleptic features due to the content of adipocytes, blood vessels, connective tissue, chondrocytes, nerves, etc. [46]. In the scaffold-based method, myoblast cells are propagated and then anchored in a bioreactor to substrate or scaffold for proliferation and differentiation [47]. Moreover, the scaffold is needed to bind and effectively utilize the nutrient content and oxygen uptake [48]. The scaffold ideal for in vitro meat should originate from non-animal sources and have a large surface for suitable growth and anchoring of cells, proper nutrient diffusion and capacity z well separate from cultured meat [49]. Moreover, it should be edible, degrade or be removable before consumption, biodegradable and non-toxic. For cultured meat, thin 3D cultures anchored in edible scaffolds can be utilized to form processed meats (nuggets, burgers, sausages).

To date, various natural polymers such as collagen, cellulose [50], chitin, amylose, textured soya [47], gelatine–soymilk mix [51], gelatine [52], and synthetic as poly (L)-lactic acid, polyglycolic acid, and polyurethanes have been used as a scaffold.

The scaffold also imposes the cellular structure of the final product to mimic the taste and texture of meat as best as possible. An ideal scaffold should be composed of edible polymers, polypeptides, hydrocolloids, or lipids [53]. If scaffolds are composed of bio polymers, these should degrade into molecules with favorable organoleptic properties. In that way, Modern Meadow in 2015 produced dehydrated, edible, high-protein ‘steak chips’, consisting of cultured muscle cells combined with a hydrogel. The development of an edible scaffold will improve the process and avoid damage during removing cell structure from scaffold. To date, the majority of cultured meat products, such as meatballs, nuggets, and ground beef do not possess ideal scaffolding architecture.

## 4. Cultivated Meat and Environment—Short Summary

With growing interest in the impact of livestock farming on the environment, a number of questions have arisen as to whether and how it will be affected by cellular meat production. In the first years, when animal-derived additives to the medium were used in the experimental stages of cell culture, huge amounts of disposable utensils and laboratory equipment were used, which raised concerns that this process would not be environmentally compatible with classic meat production. Recent years and tremendous progress in many stages of cell meat production have brought new pro-environmental solutions, which is confirmed by a number of scientific articles and independent reports. Moreover, climate changes cause long-term droughts to lead to reduced food production, food shortages, and increased food insecurity. Scientists say that cell-based foods can significantly reduce the amount of water needed to produce food, help solve global food droughts and shortages, and provide a long-term safe solution. Tuomisto et al. (2011) [40] in their study made the comparison of in vitro meat to the traditional production of beef, sheep, pork, and poultry. He noticed that to produce 1000 kg of meat by the cell culture method, 26–33 GJ of energy, 367–521 m^3^ of water, 190–230 m^2^ of land, and emits 1900–2240 kg CO_2_ equivalent of GHG are used. When analyzing the results for traditional livestock farming and meat production in Europe, the author shows that the new method mentioned above needs about 7–45% less energy (poultry). GHG emission is also lower (even up to 96%). The water consumption depends on the type of end product used in the comparison. What is not surprising, however, is that land use is reduced by 99%. The newest complex analysis showed a less negative impact of in vitro meat production on the environment, in comparison to traditional meat production of beef, pork, and chicken (Table 1).

## 5. Legislation

In many countries, there are no clearly defined regulations for both, the production and sale of cellular meat. Individual countries, in which the industrial production of cellular meat is already operating, approached the subject comprehensively, creating a number of legal regulations ensuring the marketing of a product that is safe for the final consumer. Recent months abounded with reports about the involvement of more public figures, the largest companies in the meat industry in subsequent caraway meat projects, have caused the institutions responsible for developing legal regulations in the European Union to start a loud debate and work on regulations allowing for the industrial production of this kind of product and its safe sale on EU markets and outside the EU. The following review focuses on European, US, and selected country regulatory frameworks.

### 5.1. Europe

In Europe, generally, food products that have been produced by cell culture and/or which contain tissues or cells, whether plant, animal, or fungal, are under the EU Novel Food Regulation [54]. Therefore, in vitro meat would have to go through an appropriate, formal path approving it on the market, and obtain appropriate approval from the European Food Safety Authority (EFSA). It is still not fully specified what kind of tests/approvals (in terms of food safety, declared ingredients, or nutritional value) would be required for this type of product.

The formal way of issuing food permits in the European Union is described in detail in Regulation (EC) No. 178/2002 (known as the General Food Law, GFL) [55]. This document sets out rules and procedures for Food Business Operators (FBOs). In vitro meat, before it will be sold in the EU, must, in accordance with the EFSA safety guidelines, obtain the appropriate authorization from the European Commission. This is due to the fact that this body, as its overriding objectives, primarily focuses on the safety of human health through food safety throughout the entire supply chain, in line with the “farm to fork” formula. Therefore, each element of the food production supply chain must demonstrate that the product is safe and does not endanger the consumer in any way.

Cellular meat is considered a novel food for which specific requirements are laid down in Regulation (EU) 2015/2283, also known as the Novel Food Regulation (NFR) [54] The entity responsible for placing it on the market must submit an appropriate application to the European Commission. Then, EFSA, based on the submitted documents (including scientific documents), prepares a risk assessment. This entire procedure is designed to ensure the highest level of food safety. The mentioned scientific documentation is part of the application for novel foods. This is the scientific evidence describing the properties of, for example, nutritional, potential allergens, and nutritional values. Additionally, the evidence related to the toxicological aspect of the product is analyzed in detail [54]. The European Commission takes the final decision on placing a novel food product in the European market. The outcome of this decision depends on the scientific documentation, EFSA opinion, as well as environmental and ethical aspects. Regardless of the classification of cured meat, it is also subject to the general rules applicable to each type of food, such as traceability, nutrition information, etc. [55].

Despite the positive approval of the European Commission, cellular meat, its production, and sale will be controlled by bodies selected for each member state. Quite an interesting legal issue is the nomenclature itself and the classification of cell meat as meat. In the current Food Information to Consumer Regulation [56], the definition of meat does not include meat produced using cell culture methods. This, in turn, may require that the product labeling contains specific information about the type of meat and how it was produced, so as not to mislead the consumer. In addition to the aforementioned legislation, regulations related to genetically modified organism [57,58] products may also apply to cellular meat. This is due to the fact that many innovative cell meat products use genetically modified stem cells. Unfortunately, due to the small number of scientific reports on the impact of this type of product on the human body, obtaining a license to sell such meat will be extremely difficult. Recent months show that the interest in developing detailed and clear regulations for the production and sale of cellular meat in the EU is one of the priorities of European legislation. A number of associations of companies working on the concept of cellular meat strongly lobby the need to approve this type of regulation as soon as possible, which will allow the commercial sale of this type of product, not only in countries where the law has recently allowed it.

### 5.2. USA

Regulatory management of US cell-based meat is complex. In the USA, food oversight is carried out by two agencies: the Food and Drug Administration (FDA) and the US Department of Agriculture (USDA) [59]. The FDA is responsible for eggs, dairy products, and seafood. The USDA controls the production of meat and poultry. The main document under which USDA inspections take place is the Federal Meat Inspection Act (FMIA). It should be emphasized that in many cases the FDA and USDA requirements for products are different. This made it necessary to establish close cooperation and understanding between the two organizations. In 2018 these two organizations pledged to prepare common, uniform regulations for cell meat. The supervision of cell culture, cell banks, harvesting, and isolation of cells, i.e., stages until the product is obtained from bioreactors, has been established by the FDA [60]. USDA is responsible for the subsequent production steps, including labeling. The contract itself was signed a year later, in 2019, and is a set of clear, detailed requirements for both the production and sale of cellular meat and products derived from it [59]. Importantly, in terms of general requirements for the final product, exactly the same rules apply as for any traditional meat product. However, there are still unregulated areas, such as culture media. The medium itself is the environment, the material in which the final product is located, which may cause some contamination with special ingredients/additives. For this reason, there is more and more talk about the need for standardized medium labeled as “food grade” [61]. This type of medium would not pose a health risk to the consumer. Another, but difficult-to-implement solution may be the introduction of obtaining a permit/approval for each additive used in various types of media. Such a process would have to be preceded by a series of necessary tests, which in turn is time-consuming and costly.

### 5.3. Canada

In Canada, cultured meat and seafood have been classified as a “novel food” which requires provide particular information (tests to confirm that the product is safe to eat, as well as information on allergens, levels of chemical contaminants, etc.) in the approval application prior to introduction to market [62]. The full product approval is three-part and includes: (i) a note of non-objection to human use of the food as part of the novel food evaluation process, (ii) an evaluation prior to the introduction on the market of a novel animal feed (due to possible overlaps in supply chains) and irrespective of whether the product is used as animal feed; and (iii) an environmental estimation is in line with the provisions for the application of new substances. Obtaining all three permits is necessary to sell these products in Canada. A separate legal issue is genetically engineered (GE) [62] cell meat, which will most likely be classified as a “genetically modified” product, due to the broad definition of this type of product, including any “alteration [to] the inherited characteristics of a microorganism, plant or animals by means of deliberate manipulation”. This definition includes classic methods such as conventional multiplication and mutagenesis as well as newer methods such as rDNA technology or gene editing (not limited only to the inter-species transfer of genetic material). This, in the case of cell meat, may require the producer to supply information on the complete composition of the product to assess whether the cell meat is nutritionally comparable to the meat analog, but also evidence that the food does not contain toxins or allergens inserted by genetic modification [62].

### 5.4. Asia Pacific

Asian countries reacted extremely quickly to the dynamic development of the cellular meat market by introducing comprehensive legal regulations allowing for the production and sale of ready-made products. In two countries (Singapore and Japan) significant progress has been made with regard to the regulation of cured meat.

#### 5.4.1. Singapore

In 2019, the Singapore Food Agency (SFA) has introduced guidance on the safety estimation requirements of novel foods, containing detailed information requirements to be presented for the approval of cultured meat [63]. Producers are required to conduct and submit safety estimation of protein due to potential food safety risks, associated with allergenicity, toxicity, the safety of production methods, and dietary exposure arising from intake. They must also provide detailed information about the materials and reagents used in production processes and how technology is controlled to prevent food safety risks (SFA). Currently, the SFA evaluates applications on an individual basis. A unique event on a global scale was the approval (1 December 2020) by the SFA sale of cultivated chicken bites produced by Eat Just Inc. according to the aforementioned framework. Actually, SFA discusses with local meat and seafood startups about the future approval of their products and is open to begin cooperating with companies in the initial stages of R&D and product development. What is also important are labeling requirements. Companies selling pre-packaged alternative protein products in Singapore will be required to label the product with qualifying names such as “mock”, “cultured” or “plant-based” to mark their origin, so that consumers may make a choice consciously whether to consume these products.

#### 5.4.2. Japan

Theoretical, cultured meat (depending on the production method) should be subject to existing legislation in Japan and may not need pre-market estimation or acceptance. Parallelly, the Japanese government is working on developing a specific regulatory background to properly shape the market while establishing food safety and consumer assent. Producers’ groups create industry standards and cooperate with the legislator to initiate a process that intensifies consumer confidence. In April 2020, with the government’s initiative, the Ministry of Agriculture, Forestry, and Fisheries (MAFF) initiated the Food Technology Research Group, which consists of more than 100 companies. It proposes to support the food industry and intensify Japan’s food security through modern technology. The second major government activity is the Japanese Association of Cellular Agriculture (JACA) [64], a collaboration between industry, university, and government to establish rules for cultured meat, dairy products and eggs to contribute to their commercialization in Japan. JACA is directed by the Center for Rulemaking Strategy (CRS) at Tama University, and includes 30 food companies in Japan. The CRS focuses on designing principles (law, industry norms, self-regulatory guide, etc.) for new technologies and significant ideas to be implemented in society. Japan’s Ministry of Health this summer began the regulatory approval process for cultivated meat. As cultivated meat companies around the world await regulatory approval to commercialize their genuine cell-grown meat, the Japanese government has begun an approval process for the country. The Ministry of Health, Labor and Welfare of Japan is to set up a team of experts to study the safety of cell cultured meat and its production process. A team of experts, in an effort to decide what regulations may be necessary, will be formed this fiscal year to investigate whether there are hazards in the cultivation process that could adversely affect human health. This is one of the first steps toward the eventual commercialization and industrialization of cell-cultured meat in Japan.

## 6. Conclusions

The developing meat market and its growing needs force dynamic changes not only in the entire supply chain, but also in the search for various types of alternatives to classic products of animal origin. Today’s livestock production, necessary for the production of meat in its classic form, is still not sustainable and constitutes a huge environmental burden [4]. What additionally forces us to look for alternatives is the increase in feed production costs related to the difficult access to raw materials in many countries caused by climate change (droughts), the situation in Eastern Europe (war in Ukraine), unstable, high fuel and electricity prices mean that the final costs of producing classic food of animal origin are getting higher. It should be emphasized that, apart from the environmental footprint, various ethical (mainly animal welfare) and health issues (related not only to attempts to link meat consumption to certain disease entities, but mainly to the risk of such zoonoses caused by *Salmonella* spp. and *Campylobacter* spp., *E. coli*, or the growing phenomenon of drug resistance caused by the excessive use of antibiotics in animal husbandry) have led to growing criticism of traditional meat. In vitro meat is an alternative source of protein today that still faces a number of challenges. These are mainly obtaining the appropriate nutritional values and the naturalness of the product (mainly in terms of the medium and its additions). Products of this type reduce the need to increase the breeding of slaughtered animals, and reduce the microbiological risk [7], whether related to the increasing drug resistance of antibiotics commonly used in veterinary medicine or as growth stimulants (still allowed in some countries). It is worth emphasizing that this type of meat production has a much lower negative impact on the environment, which is crucial in the case of trends related to sustainable production.

The cultivated meat market (otherwise: cellular, in vitro, synthetic, etc.; the nomenclature is just emerging) is a nascent industry for which 2020 was a breakthrough, when the cultivated chicken product presented by Eat Just made its debut on the restaurant menu in Singapore after the national food agency approved the right to sell it. The starting point for the market presence of cellular meat can be considered in 2013, when the first burger from cultivated meat was presented during a press conference in London, the creation of which cost approximately $330,000. The number of startups focused on the development of cultivated meat (and the required carriers for cell culture, supplements, and methods of their production) is growing every year, mostly, around Europe, Asia, and USA (Table 2). The cultured cell meat is ethically produced as the livestock is not used in the production processes except for the collection of the initial cells required for cell culture. There are now about 100 companies worldwide that develop cultured meat ingredients, services, and end products, compared to just four in 2016. As of today (2022), the price of cell culture meat has fallen from $330,000 to about $9 or $9.80 for a burger. Prices are falling as the production scale improves and materials cost less. Nevertheless, meat grown in a laboratory is still “significantly more expensive” than a burger you can buy at a grocery store or restaurant.

What has been observed recently is that business-to-business companies are emerging that cover the entire supply chain in the cultivated meat industry, including low-cost cell culture media, bioreactors, scaffold materials, and cell lines. This is probably one of the trends that will be also observed during the next years. Due to the growing interest in other, unpopular species of cellular meat obtained from animal species unusual for the food industry, scientists face challenges related to the development of cell isolation and culture protocols, as well as the development of stable cell lines on which further production will be based. It should be emphasized that such cultures may require completely different types of media and additives used in them. In addition, at the next stages of production, related to the development of the final product, the supply to the consumer may require the development of new additives responsible for the texture, taste, smell, color, etc. In the case of cellular media, the constant challenge will be to develop a medium that will meet all legal requirements for approval for use in food, it will be at an acceptable price and generally available. Another challenge for scientists will be to develop safe, natural additives that give the right taste, smell, color, and texture. It seems that the development of natural replacements for growth stimulators (e.g., based on phytobiotics or designed single proteins) will also continue to be a key research topic. In addition, already at the industrial stage, the creation of entire production lines allowing for the rapid production of larger amounts of cellular meat seems to be still a topical topic. Increasingly, the issue of effective recovery (modeled on the phenomenon of dialysis) of culture media is raised. However, the pace of development of all branches related to the mobile industry depends on one issue, not entirely dependent on the entire industry—legal regulations. However, it seems that a milestone made towards the industry in the USA will accelerate the work on the development of legal regulations for the production and marketing of cellular meat in countries where more and more companies in this industry are established every year.

In many countries (mainly Europe), the development and implementation of technology using molecular biology methods (genetic modification) to immortalize the cells will be a major challenge. Therefore, it should be expected that the next years of research will be largely focused on the safety of the product for the consumer and adjusting to what the consumer is able to accept.

## Figures and Tables

**Table 1:** The effect of cultivated meat production on selected environmental aspects: comparison with traditional meat production for three types of meat. (based on CE Delf TEA report, November 2021).

	Cultured Meat Compared to Traditional Chicken	Cultured Meat Compared to Traditional Pork	Cultured Meat Compared to Traditional Beef
Carbon footprint	17% reduction	52% reduction	92% reduction
Land Use	63% reduction	72% reduction	95% reduction
Air pollution	29% reduction	49% reduction	93% reduction

**Table 2:** Selected companies identified on the cultivated meat market from 2019–2022. Red meat means beef or/and pork, other means other types of meat/cells/media, etc.

Company Name	Country	Product/Type of Meat
Cell Farm Food Tech/Granja Celular S.A.	Argentina	Other
Magic Valley	Australia	Red Meat
Vow	Australia	Kangaroo
Heuros	Australia	Petfood
Because Animals	Austria/USA	Petfood
Appleton Meats	Canada	Red Meat
Meatleo	Canada	Red Meat
Avant Meats Company Limited	China	Fish/Seafood
CellX	China	Red Meat
Mewery	Czech Republic	Red Meat
Gourmey	France	Poultry
Avant Meat	Hong Kong	Seafood
Clear Meat	India	Chicken
SuperMeat	Israel	Chicken
Future Meat Technologies Ltd.	Israel	Poultry
Biofood systems Ltd.	Israel	Other
MeaTech 3D	Israel	Poultry/Red Meat
Savor Eat	Israel	Red Meat
Meatech (Peace of Meat)	Israel/Belgium	Chicken (foie gras)
Aleph Farms Ltd.	Israel	Red Meat
Integriculture	Japan	Poultry/Foie gras
Integriculture	Japan	Chicken
MosaMeat	Netherland	Red Meat
Meatable	Netherland	Red Meat
Ants innovate	Singapore	Red Meat
Gaia Foods	Singapore	Red Meat
Shiok Meats	Singapore	Seafood
Eat just (Good Meat division)	Singapore /USA	Chicken
Mzsansi Meat	South Africa	Poultry/Red Meat
Mogale Meat	South Africa	Antelope
Cubiq Foods	Spain	Other
Biotech Food	Spain	Other
Cubiq Farms	Spain	Other
Mirai Foods	Switzerland	Red Meat
Biftek	Turkey	Red Meat
Higher Steaks	UK	Other
Higher Steaks	UK	Red Meat
Hoxton Farm	UK	Other
Memphis Meat	USA	Red Meat
Eat Just	USA	Poultry
Finless Food Inc.	USA	Poultry/Red Meat
Balletic Foods	USA	Other
Lab Farm Foods	USA	Poultry/Red Meat
Orbillion Bio	USA	Red Meat/Other
Blue Nalu	USA	Seafood
Hampton Creek (Eat Just)	USA	Poultry
Finless Food	USA	Poultry/Red Meat/ Seafood
Institute of the future	USA	Other
Fork and Goode	USA	Other
Bond Pet Food	USA	Petfood
Upside Food	USA	Chicken
Mission Barns	USA	Other
Paerlita Food	USA	Other
Wild Earth	USA	Petfood
Wild Type	USA	Seafood
Motif FoodWorks	USA	Red Meat
New Age Meats	USA	Red Meat

## Data Availability

Data is contained within the article.
